# Ultra-processed Food and Obesity: What Is the Evidence?

**DOI:** 10.1007/s13668-024-00517-z

**Published:** 2024-01-31

**Authors:** Samuel J. Dicken, Rachel L. Batterham

**Affiliations:** 1 Centre for Obesity Research, Department of Medicine, University College London (UCL), London, WC1E 6JF UK; 2 Bariatric Centre for Weight Management and Metabolic Surgery, University College London Hospital (UCLH), London, NW1 2BU UK; 3 National Institute for Health Research, Biomedical Research Centre, University College London Hospital (UCLH), London, W1T 7DN UK

**Keywords:** Ultra-processed food, Obesity, Dietary guidelines, NOVA, Diet, Public health

## Abstract

**Purpose of Review:**

Obesity is a growing global healthcare concern. A proposed driver is the recent increase in ultra-processed food (UPF) intake. However, disagreement surrounds the concept of UPF, the strength of evidence, and suggested mechanisms. Therefore, this review aimed to critically appraise the evidence on UPF and obesity.

**Recent Findings:**

Observational studies demonstrate positive associations between UPF intake, weight gain, and overweight/obesity, more clearly in adults than children/adolescents. This is supported by high-quality clinical data. Several mechanisms are proposed, but current understanding is inconclusive.

**Summary:**

Greater UPF consumption has been a key driver of obesity. There is a need to change the obesogenic environment to support individuals to reduce their UPF intake. The UPF concept is a novel approach that is not explained with existing nutrient- and food-based frameworks.

Critical analysis of methodologies provides confidence, but future observational and experimental research outputs with greater methodological rigor will strengthen findings, which are outlined.

**Supplementary Information:**

The online version contains supplementary material available at 10.1007/s13668-024-00517-z.

## Introduction

Obesity is a chronic, complex disease, defined by the World Health Organisation (WHO) as an “excess accumulation of fat mass that significantly impairs health” [1]. Approximately two billion adults worldwide live with overweight (a body mass index (BMI) ≥ 25 kg/m^2^) [1], and over 650 million with obesity ( ≥ 30 kg/m^2^). Worryingly, prevalence has increased six-fold in just a few decades, up from 105 million in 1975 [2].

Obesity reduces quality of life and increases the risk of non-communicable disease (NCD), morbidity and all-cause mortality (ACM) [3, 4]. Globally, obesity costs nearly $2 trillion/year, with direct and indirect costs, including on healthcare and economic productivity. These figures are expected to rise to $4 trillion by 2035, nearly 3% of global gross domestic product [5, 6].

Understanding the causes of obesity is of paramount importance for prevention and treatment, being the focus of a recent Royal Society Scientific meeting [7]. Individual, social and environmental factors all influence weight regulation. However, general scientific consensus points towards recent environmental changes driving obesity onset [8], with complex interactions between individual-level factors and socio-environmental determinants driving an energy surplus and excess adiposity [9].

Diet is fundamental for weight management. Maintaining a healthy body weight and preventing weight gain are key features of national dietary guidance [10], which to date has been conceptualised by food groups and nutrients. However, research in the last 5 years has provided new insights into the relationship between diet and obesity. Indeed, one major recent environmental change has been to the food environment, with increased availability and consumption of ultra-processed food (UPF). This has shifted the types of food and drink available and consumed [11, 12•], with potentially important impacts on obesity.

Literature on UPF has quickly grown. Before 2018, a PubMed search for ‘ultra-processed’ retrieved 137 papers. By November 2023, there were 1558 papers. The aim of this review is to critically appraise the growing body of human evidence on UPF and obesity, discussing potential mechanisms, methodological rigor and implications for future research and policy.

## Literature Search

**Fig. 1:**
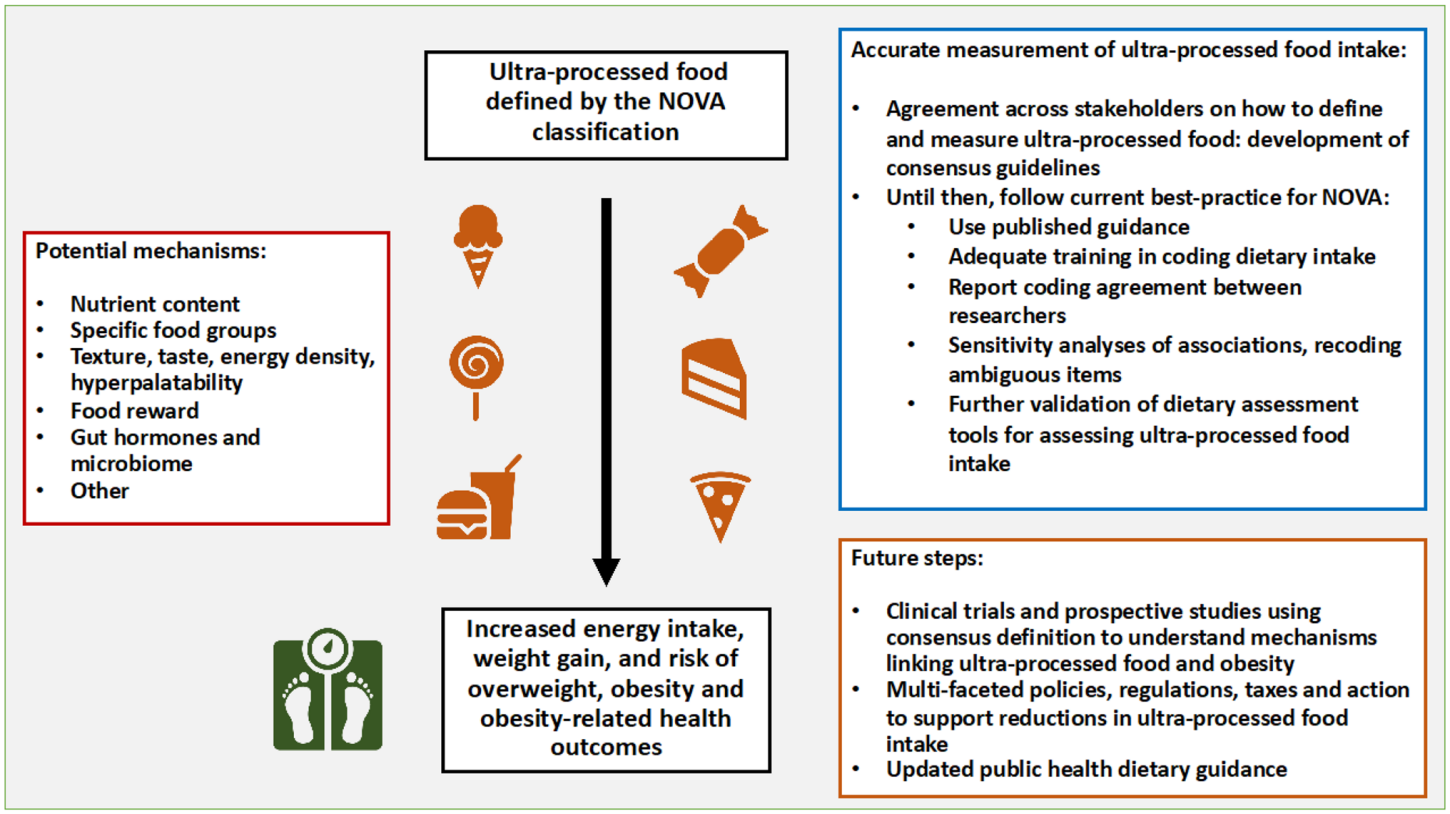
Summary of review findings regarding ultra-processed food and obesity

To ensure a broad scope, a search of PubMed for ultra-processed and ‘ultra-processed AND obesity’ was conducted. Peer-reviewed articles in English, primarily from 2018 to 2023, including systematic reviews, narrative reviews, clinical trials and observational studies were considered if they assessed dietary intake according to NOVA with nutritional characteristics, adiposity-related outcomes or eating behaviour. Papers considering methodological aspects of assessing NOVA intake and policy implications relating to UPF intake were also considered. Further papers were obtained from peers and through checking references of citations. Retrospective cohort studies, lab or rodent studies were excluded. Papers of notable mention prior to 2018 were included, as well as wider relevant papers. Additional systematic reviews, narrative reviews and prospective observational studies identified during the search are provided in the Supplementary Materials. In total, 132 articles were included. The findings from this review are summarised in Fig. 1.

## Food Processing

Food processing (any procedure altering the natural state of food) is fundamental for food preparation. Basic processing techniques have existed for thousands of years and include removing inedible parts from foods, cooking, and chopping. Most foods are processed in some way before consumption. More recently, processing methods such as bottling, canning or use of additives have enabled extended shelf-life, increased safety [13] and reduced waste of food [14, 15•]. This has provided food security for millions worldwide. However, some processing methods can be deleterious for food safety and health [15•], such as trans-fat formation from hydrogenation of vegetable oils. Therefore, distinguishing between harmful, neutral and beneficial forms of processing is of public health importance.

Several classifications exist for grouping foods according to processing [16, 17]. The most frequently used (but not necessarily the most valid) is the NOVA classification (not an acronym). Conceptualised in 2010 [18], NOVA classifies food and drink into four groups based on the extent and purpose of processing: minimally processed foods (MPF), processed culinary ingredients (PCI), processed foods (PF) and UPF (Table 1) [19, 20, 21••]. NOVA considers not just the physical extent of processing, but also the purpose of processing [17]. This holistic approach to also consider the purpose of processing is a significant conceptual shift in nutrition science, contrasting with the traditional reductionist, bottom-up approach to date [22]. Of particular interest are UPFs, which are considered to contain harmful forms of processing. UPFs are defined as industrial formulations typically with five or more ingredients, using extracts of original foods. These include soft drinks, breakfast cereals and packaged snacks. Global UPF intake is high, having rapidly increased in recent decades [12•]. This is particularly so in high- and middle-income countries, with great variation in intake across sociodemographic profiles [23].

**Table 1:** The NOVA classification, from Monteiro et al. [20] and Steele et al. [24]

**NOVA group**	**Purpose**	**Types of processing and examples**
**Minimally processed foods**	To preserve foods and make it possible to store them and, sometimes, also to reduce the stages of food preparation (cleaning and removing inedible parts), to facilitate their digestion, or to render them more palatable (grinding or fermentation).	Unprocessed foods altered by industrial processes such as removal of inedible or unwanted parts, drying, crushing, grinding, fractioning, roasting, boiling, pasteurisation, refrigeration, freezing, placing in containers, vacuum packaging, non-alcoholic fermentation and other methods that do not add salt, sugar, oils or fats or other food substances to the original food. The purpose of these processes are to preserve foods, make them suitable for storage, safe to eat, edible to eat or more pleasant to eat [24]. Minimally processed foods include fresh, frozen or dried fruits and vegetables; grains; legumes; meat, poultry, fish; eggs; milk; fruit or vegetable juices (with no added sugar, sweeteners or flavours); flakes or flour made from corn, wheat, oats; seeds (with no added salt or sugar); herbs and spices, plain yoghurts; tea, coffee and water.
**Processed culinary ingredients**	To obtain ingredients used in the home and in restaurant kitchens to prepare, season and cook unprocessed or minimally processed foods, to create varied and enjoyable dishes.	Substances obtained directly from minimally processed foods or from nature by industrial processes such as pressing, centrifuging, refining, extracting or mining. Examples include vegetable oils; butter and lard; sugar and molasses; honey and starches from corn and other plants and salt.
**Processed foods**	To prolong the durability of minimally processed foods and make them more enjoyable, by modifying or enhancing sensory qualities.	Products made by adding salt, oil, sugar or other processed culinary ingredients to minimally processed foods, using preservation methods such as canning and bottling or for breads and cheeses, non-alcoholic fermentation. Examples include canned or bottled vegetables and pulses in brine; salted nuts and seeds; salted, dried, cured or smoked meats or fish; canned fish; fruit in syrup and freshly made unpackaged or artisanal breads and cheeses.
**Ultra-processed foods**	To make long-lasting, readily available and accessible (ready to eat) hyperpalatable and highly profitable (using low-cost ingredients) branded products, often consumed as fast food, snacks or desserts [24], to displace all other food and drink groups [20].	Formulations of ingredients mostly of exclusive industrial use, which result from a series of industrial processes. Many processes require sophisticated equipment and technology. Processes include fractioning whole foods into substances, chemical modifications of substances, assembly of unmodified and modified food substances using industrial techniques such as hydrogenation, hydrolysation, extrusion, moulding and pre-frying, frequent application of additives whose function is to make the final product palatable or hyper-palatable (‘cosmetic additives’) and sophisticated packaging, usually with synthetic materials [20].
		Ingredients often include sugar, oils and fats and salt, which are usually used in combination and substances that are sources of energy and nutrients but of no or rare culinary use. These include high-fructose corn syrup, hydrogenated or interesterified oils and protein isolates; cosmetic additives such as flavours, flavour enhancers, colours, emulsifiers, sweeteners, thickeners, and anti-foaming, bulking, carbonating, foaming, gelling and glazing agents and additives that prolong shelf-life, protect the original properties of the product or prevent microorganism proliferation.
		Examples include soft drinks; sweet/savoury packaged snacks; chocolate; ice cream; mass-produced packaged breads; margarines; biscuits, pastries and cakes; breakfast ‘cereals’, ‘cereal’ and ‘energy’ bars; ‘energy’ drinks; milk drinks, ‘fruit’ yoghurts and ‘fruit’ drinks; ‘cocoa’ drinks; ‘instant’ sauces; infant formulas and follow-on milks; ‘health’ and ‘slimming’ products such as meal replacement shakes and powders. Ultra-processed foods also include many ready-to-heat products including pre-prepared pies, pasta and pizza meals; chicken and fish ‘nuggets’ and ‘sticks’, sausages, burgers, hot dogs and other reconstituted meat products and powdered and packaged ‘instant’ soups, noodles and desserts.

## Evidence for UPF and Obesity

Evidence linking UPF intake with weight management and obesity is largely from observational studies. A number of reviews have summarised results. These all indicate that with increasing intake of UPF, there are increased risks of weight gain, overweight and obesity. In adults, four meta-analyses show that greater UPF intake is associated with increased risks of overweight (odds ratio (OR), 1.36 (95% confidence interval (CI), 1.14–1.63); OR, 1.02 (95%CI, 1.01–1.03); OR, 1.36 (95%CI, 1.23–1.51)), obesity (OR, 1.55 (95%CI, 1.36–1.77); OR, 1.26 (95%CI, 1.13–1.41); OR, 1.51 (95%CI, 1.34–1.70)) and overweight/obesity (OR 1.39 (95%CI, 1.29–1.50)), in a dose–response manner [25–28]. Meta-analyses also show increased risks of abdominal obesity or increased waist circumference (WC) (OR, 1.41 (95%CI, 1.18–1.68); OR, 1.39 (95%CI, 1.16–1.67); OR, 1.49 (95%CI, 1.34–1.66)) [26–28]. However, these mainly consist of cross-sectional studies (9/12 and 13/14 in two meta-analyses [27, 28]). All eight prospective studies in one systematic demonstrated positive associations with abdominal obesity and obesity [29]. A meta-analysis of two prospective studies also demonstrated increased risks of overweight/obesity (relative risk, 1.23, 95%CI: 1.11–1.36)) [28]. Numerous other prospective studies report increased risks of weight gain and obesity with increasing UPF consumption, in a dose-response manner (see Supplementary Materials), as well as other systematic/non-systematic reviews, largely citing the same evidence [30].

In children and adolescents, systematic reviews demonstrate increased risks of overweight, obesity and elevated WC with greater UPF intake [31, 32]. However, findings are less conclusive, and most studies are from Brazil. In one systematic review, 7/13 studies demonstrated increased risks of overweight/obesity [31]. In another, 4/5 prospective studies demonstrated a positive association with obesity or adiposity parameters, but 5/5 cross-sectional studies did not [32]. A small number of other prospective studies in children do not suggest an increased risk with greater UPF intake (Supplementary Materials).

Experimental evidence also supports a role of UPF in obesity, by promoting greater energy intake and weight gain. In a randomised, controlled, crossover metabolic ward trial by Hall and colleagues, 20 adults consumed an ad libitum, 2-week minimally processed diet and an ad libitum, 2-week ultra-processed diet [33••]. Participants consumed 500 kcal/day more on the UPF diet compared with the MPF diet, gaining 0.9 kg on the UPF diet, and losing 0.9 kg on the MPF diet [33••]. A single-day ad libitum feeding trial also found greater energy intake from a day of UPF, compared with a day of MPF [34].

Less well evidenced are the associations of other NOVA groups (MPF, PCI and PF) with obesity. MPF intake has been inversely associated with weight gain, WC, overweight and obesity and resulted in weight loss in Hall et al. [33••], whereas PFs have demonstrated a neutral association [35–38].

In summary, greater UPF intake is associated with deleterious impacts on weight management, which is not observed with other NOVA groups. But, is ultra-processing per se to blame? Or, can the effects be explained by existing knowledge of diet and obesity?

## Mechanisms: What Drives the Effect?

A range of plausible mechanisms exist by which ultra-processing may promote energy overconsumption and weight gain. This includes their nutrient and energy content, displacement of healthy food groups, matrix degradation, altered texture, taste, satiety and additive content [39–41], dysregulation of mechanisms of weight regulation and behavioural and environmental aspects such as hyperpalatability, marketing, low cost, portion size, availability and convenience [42••, 43]. Some academics have even argued that UPFs have addictive potential, but this is debated [44].

It has been argued that the main determinants of chronic disease risk are captured within existing nutrient profiling models [45], and current public guidance is sufficient for health [46]. Indeed, UPFs tend to have higher energy densities and lower nutrient densities than MPFs [47, 48], and high-UPF diets are associated with greater intakes of energy, free sugars, fat and saturated fat and lower intakes of fibre, protein and some micronutrients [49]. High-UPF diets also contain less fruit, vegetables, beans and legumes than low-UPF diets [49]. Therefore, the effects of UPF could be explained by nutrient- or food-based factors known to influence weight management. However, both experimental and observational evidences do not support this. In Hall et al. [33••], the UPF and MPF diets were matched for presented energy and nutrients (carbohydrate, sugar, fat and fibre). Yet, there were divergent changes in energy intake and weight. However, the UPF and MPF diets differed for added-to-total sugar (54% vs. 1%, respectively), insoluble-to-total fibre (16% vs. 77%, respectively), saturated-to-total fat (34% vs. 19%, respectively) and omega-3-to-omega-6 fats (11:1 vs. 5:1, respectively). In observational studies, a lack of adjustment for diet (e.g. nutrients, food groups or diet quality indices) has been suggested as a limitation [50]. However, Dicken and Batterham reviewed the impact of dietary adjustments on associations between UPF and obesity outcomes in prospective studies [42••]. Most studies had performed dietary adjustments. Of the 23 significant associations between UPF intake and obesity/weight outcomes (out of 26 models), 93% (40/43) of all dietary adjustments did not alter or explain these significant associations. Since the review, additional cohort studies find increased risks independent of diet quality [37, 51–56] (Table 2). To date, 17 prospective cohort studies report 40 significant associations (out of 45 models) between UPF intake and obesity/weight outcomes in adults and children. Of these, 93% (37) were unchanged after adjustment for nutrients, food groups, diet patterns or other NOVA groups.

**Table 2:** Prospective cohort studies assessing the association between UPF intake and obesity indicators, adjusting for diet quality

**Author date**	**Location**	**Cohort**	**Sample**	**Diet assessment method**	**Outcome**	**Significant before diet adjustment (Yes/No)**	**Significant after diet adjustment (Yes/No)**	**Diet adjustments made**
Mendonça et al. [57]	Spain	Seguimiento Universidad de Navarra (SUN)	Middle-aged University graduates	FFQ	Overweight/obesity	Y	Y	Fruit and vegetables
Rohatgi et al. [58]	MO, USA	Women’s Health Center and Obstetrics & Gynecology Clinic	Pregnant females and neonates	FFQ	Gestational weight gain (kg)	Not computed	Y	Fat intake
					Neonate thigh skinfold thickness (mm)	Not computed	Y	
					Neonate subscapular skinfold thickness (mm)	Not computed	Y	
					Neonate body fat percentage (%)	Not computed	Y	
Canhada et al. [59]	Brazil	Brazilian Longitudinal Study of Adult Healt (ELSA-Brazil)	Civil servants aged 35–74	FFQ	Large weight gain (≥ 90th percentile: ≥ 1.68 kg/year)	Y	Y	Fruit and vegetables
					Large WC gain (≥ 90th percentile: ≥ 2.42 cm/year)	Y	Y	
					Incident overweight/obesity	Y	Y	
					Incident obesity	N	N	
Beslay et al. [35]	France	Nutri-Net Santé	Adults ≥ 18	24-h recall	BMI change (kg/m^2^)	Y	Y	(1) Sugar, sodium, SFAs, and dietary fibre; (2) healthy and Western dietary patterns; (3) fruit and vegetables and sugary drinks
					Overweight	Y	Y	
					Obesity	Y	Y	
Sandoval-Insausti et al. [60]	Spain	Study on Cardiovascular Health, Nutrition and Frailty in Older Adults in Spain (ENRICA) Seniors-ENRICA-1	Older adults	7-day diet history	Abdominal obesity	Y	Y	Fibre, very-long-chain omega-3 fatty acids and Mediterranean diet
Li and Shi [61]	China	China Health and Nutrition Survey (CHNS)	Adults > 20	Three 24-h recalls	Overweight/obesity	Y	Y	Fat intake and dietary pattern (traditional pattern characterised by high intake of rice, pork and vegetables, and low intake of wheat and a modern dietary pattern characterised by high intake of fruit, soy milk, egg, milk and deep-fried products)
					Central obesity	Y	Y	
Koniecnzna et al. [62]	Spain	PREDIMED-Plus	Adults aged 55–75 with overweight/obesity and metabolic syndrome	FFQ	Total fat mass (*z*-score)	Y	Y	(1) Sodium, saturated and trans fats, alcohol, fibre, glycaemic index, Mediterranean Diet; (2) changes in fruit intake; (3) changes in vegetable intake; (4) changes in fibre intake
					Visceral fat mass (*z*-score)	Y	Y	
					Android/gynoid fat ratio (*z*-score)	Y	N	
Cordova et al. [36]	Multi-national (nine countries)	European Prospective Investigation into Cancer and Nutrition (EPIC)	Adults aged 25–70	FFQ, s-FFQ and diet record	Weight gain (kg)	Y	Y	Mediterranean diet
					Overweight/obesity	Y	Y	
					Obesity	Y	Y	
Chang et al. [63]	England	Avon Longitudinal Study of Parents and Children (ALSPAC)	Children	Three-day food diary	BMI change (kg/m2)/year	Y	Y	(1) Fruit and vegetables; (2) saturated fat, sugar, fibre, and sodium
					Fat mass index change (kg/m^2^)/year	Y	Y	
					Lean mass index change (kg/m^2^)/year	N	N	
					Body fat percentage change (%)/year	N	N	
Costa et al. [64]	Brazil	Pelotas-Brazil 2004 Birth Cohort	6–11-year-olds	FFQ	Fat mass index (kg/m^2^)	Y	Y	Food sources other than ultra-processed food
Wang et al. [51]	USA	Nurses’ Health Study II (NHSII) and the Growing Up Today Study (GUTS I and II)	Mother-child (aged 7–17 years at enrolment) pairs	FFQ	Offspring Overweight	Y	Y	Maternal Alternative Healthy Eating Index 2010, offspring consumption of ultra-processed foods
					Offspring Obesity	Y	Y	
					Offspring BMI percentile	Y	Y	
González-Palacios et al. [53]	Spain	Prevención con Dieta Mediterránea (PREDIMED)-Plus	Adults aged 55–75 without baseline cardiovascular disease	FFQ	Weight	Y	Y	Mediterranean diet
					BMI	Y	Y	
					WC	Y	Y	
Pan et al. [52]	China	China Health and Nutrition Survey (CHNS)	Adults ≥ 18	Three 24-h recalls	Central obesity	Y	Y	Protein, fat, carbohydrate and sodium
Tan et al. [37]	South Korea	Health Examinees (HEXA)	Adults ≥ 40 years	FFQ	Obesity	Y females/N males	Y females/N males	Carbohydrate, protein, fat, processed food, unprocessed or minimally processed food, and processed culinary ingredients.
dos Santos et al. [55]	Brazil	NutriNet-Brasil	Adults ≥ 18	Three non-consecutive NOVA 24-h screener recalls	BMI gain (%)	Y	Y	Unprocessed or minimally processed whole plant foods
					BMI increase (≥ 5%)	Y	Y	
Pang et al. [54]	USA	Coronary Artery Calcification in Type 1 Diabetes (CACTI)	T1DM and non-diabetic controls	FFQ	Weight	Y	Y	Unprocessed food (i.e. mainly fruits and vegetables)
					WC	Y	Y	
					BMI (kg/m^2^)	Y	Y	
					Overweight	Y	Y	
					Obesity	Y	Y	
Pan et al. [56]	China	Chinese Food Consumption Survey 2017–2020	Adults ≥ 18	Three 24-h recalls	Overweight/obesity	Y	N	Protein, fat, carbohydrate, vitamin A, vitamin C, calcium, and sodium
					Overweight	Y	N	
					Obesity	N	N	

*BMI* body mass index, *FFQ* food frequency questionnaire, *UPF* ultra-processed food, *WC* waist circumference

It could be that the adverse effects of UPFs are driven by specific products known to impact weight management [50], such as sugar-sweetened beverages (SSB) [65]. Also, given the heterogeneity in nutrient content, and that some short-term RCTs do not necessarily suggest detrimental impacts of all UPFs on weight management (e.g. isolated proteins [66]), some UPFs have been considered to be ‘healthy’ [47, 67]. This may question an overall effect of ultra-processing. If ultra-processing per se does have an independent influence on weight, the same food with different processing should have contrasting associations with obesity (e.g. MPF vs. UPF dairy, fruit or meat products).

To date, few studies have compared the association of multiple UPF sub-groups, or like-for-like foods across NOVA groups, with obesity. In those that have, there are inconsistent associations between each UPF sub-group and obesity, and associations do not appear to be explained by specific UPFs. In France, UPF drinks (including SSBs and artificially sweetened beverages (ASBs)), dairy products, fats and sauces and meat, fish and egg products were each associated with increased risks of overweight and obesity [35]. Ultra-processed starchy foods and breakfast cereals were associated with increased risks of overweight, but not obesity [35]. These food groups were not significantly associated with obesity outcomes in their non-ultra-processed form, except for meat, fish or egg products (e.g. smoked meat or hams). Ultra-processed salty snacks, sugary products and fruit and vegetables were also not associated with overweight or obesity. In Spain, the highest vs. lowest tertile of intake of several UPF sub-groups (dairy products, ultra-processed meats, pre-prepared dishes, snacks and fast-foods, sweets, alcoholic and non-alcoholic beverages) was associated with increased total fat mass and visceral fat mass (except sweets and non-alcoholic beverages). Only dairy was associated with increased android-to-gynoid fat ratio [62]. In another Spanish cohort, non-alcoholic beverages (instant coffee, cocoa and packaged fruit juices, not including soft drinks) were significantly associated with abdominal obesity, but other sub-groups were non-significant [60]. In Brazilian children, sweets were significantly associated with changes in WC and waist-to-height ratio, but only after adjustment for BMI. Other UPF sub-groups were non-significant [64]. In three US cohorts, maternal intake of ultra-processed breads and breakfast foods, but not other subgroups (sauces, cheeses, spreads and gravies, ultra-processed beverages, packaged sweets and desserts), was associated with increased risks of offspring overweight or obesity [51]. In Brazil, the adverse association of UPF with adiposity outcomes was not explained by SSB intake [59], and across nine European countries, the association of UPF with weight gain was not explained by soft drink consumption (but attenuated the association by one third) [36]. Currently, no systematic review has examined studies performing sub-group analyses or studies comparing associations of the same food across processing levels. Routine conduct of sub-group analyses in cohort studies will greatly enhance quantifying the potential heterogeneity in the detrimental associations of UPF with obesity.

UPFs tend to be more energy dense than MPFs or PFs [47, 48], containing unique ‘hyperpalatable’ combinations of carbohydrate, fat and salt that are not usually seen in nature [47, 68]. These factors may override homeostatic feeding mechanisms, alter taste-nutrient relationships and facilitate faster eating rates [69]. In Hall et al., nearly half (45.1%) of the increased energy intake on the UPF diet was mediated by energy density [69].

Industrial ultra-processing can lead to extensive matrix degradation, making food softer and easier to consume quickly [34]. Faster eating rates (weight of food consumed) can promote greater energy intake rates (EIR) and energy intake [34, 69]. This is particularly so when consuming energy dense foods, being associated with increased bodyweight [70]. In controlled feeding trials, the EIR of UPFs is nearly double that of MPFs, with the EIR of PFs in between (MPF, 35.5 ± 4.4; PF, 53.7 ± 4.3; UPF, 69.4 ± 3.1 kcal/min) [71]. In Hall et al., meal and beverage EIR was higher on the UPF than MPF diet, which was due to a higher UPF beverage EIR. In a crossover trial of four single ad libitum meals (hard-textured or soft-textured MPF or UPF chicken, potato, vegetables and fruit), greater energy intake was consumed at the soft-textured UPF vs. soft-textured MPF meal, and also at the hard-textured UPF vs. hard-textured MPF meal [34]. The authors reported a significantly greater eating rate in the soft-textured UPF vs. soft-textured MPF meal, but not between hard-textured meals [34]. With the greater energy density of UPFs, EIR was greater for both soft- and hard-textured UPF meals vs. the texture-matched MPF meal. Notably, the difference in energy intake between UPF vs. MPF meals was not compensated for at meals later in the day. However, the presented weight or energy content was matched between meals. In a pilot study, energy intake was non-significantly ~ 400 kcal higher on a 1-day diet of soft-textured UPF vs. soft-textured MPF matched for energy density [72]. Interestingly, energy intake was non-significantly ~ 300 kcal greater on the hard-textured MPF vs. hard-textured UPF 1-day diet. There was an effect of processing on EIR, and an interaction effect of processing with texture on energy intake [72]. However, the study was underpowered, recruiting 18 instead of the intended 60, potentially explaining the non-significance in energy intake between menus. Furthermore, only 29% and 52% of energy provided in the hard- and soft-textured UPF meals, respectively, was UPF. Most of the energy in the hard-textured UPF meal came from PF than UPF.

The unique and hyperpalatable combinations of fat, sugar and salt in UPFs [47] and use of flavourings, colours or sweeteners may alter taste-nutrient relationships and may promote weight gain through hedonic eating [73]. Food reformulation with low-calorie sweeteners may lead to inaccuracies in relaying nutrient content to the brain, as sweetness may no longer be proportional to sugar or calorie content [74]. Lower-calorie, artificially sweetened drinks can condition towards a greater brain response and liking than a higher-calorie drink with similar sweetness perception [75]. Observational data from Singapore suggests that taste-nutrient relationships are maintained across NOVA groups [76]. However, UPFs had stronger associations between fat taste and fat content and salt taste and salt content, and weaker associations between sweet taste and sugar content than MPFs [76]. The greater EIR of UPFs may also generate a greater food reward that alters gut-brain signalling, flavour-nutrient conditioning and food preference [77]. However, an exploratory study of Hall et al. found no difference in sweet or salty taste preference or taste detection thresholds after UPF or MPF diets [78]. Sweet or salty taste preference or detection was unrelated to ad libitum sugar or salt intake on either diet [78].

Distinct from energy density [69], hyperpalatable foods (HPF) may predispose to greater energy intake. In Hall et al., HPF consumption mediated 41.9% of the greater energy intake on the UPF diet [69]. In a prospective analysis, consuming a greater proportion of carbohydrate- and sodium-rich HPF at a single ad libitum buffet meal was associated with weight and body fat gain over the following year, but a positive association was not seen for consuming a greater proportion of UPF, high-energy density foods, or fat- and sodium-rich HPF at the single meal [79].

Food ‘liking’ and ‘wanting’ are distinct concepts in food choice. Evidence suggests UPFs may be ‘wanted’ more, but do not seem to be ‘liked’ to a greater extent during consumption. Before consumption, images of UPFs generate a strong appetitive drive [80], with greater motivational reactivity than MPFs or PFs [81], and greater approach motivation and intent to consume than MPFs [82]. During and after consumption, early preliminary evidence suggested that MPFs had a higher satiety potential than UPFs [83, 84]. More recently in the trial comparing a single hard- and soft-textured MPF and UPF lunch [34], self-rated pleasantness at the first bite of the meal did not significantly influence energy intake, with similar post-meal appetite ratings between lunches. Besides the hard-textured MPF which was rated as significantly less pleasant, the hard-textured UPF and both soft-textured meals were rated similarly for pleasantness. Likewise, participants in Hall et al. reported no differences in pleasantness, satisfaction, hunger or fullness between MPF and UPF diets [33••]. The underpowered pilot study also reported minimal differences in appetite ratings after UPF or MPF meals [72]. In an online virtual study asking a convenience sample to imagine taking a bite from a range of foods, whereby UPFs provided a greater desire to eat (reward), greater taste intensity and were liked more (taste pleasantness) than MPFs independent of energy density, but not more so than PFs [85]. In summary, this suggests a potentially increased wanting to consume UPFs, but similar liking to MPFs when consumed to satiety, but with different resulting energy intakes.

Lastly, UPFs may adversely impact on homeostatic mechanisms of weight regulation. In Hall et al., active ghrelin (appetite stimulating hormone) and adiponectin decreased, and peptide YY (PYY) (satiety hormone) increased, after the MPF diet. In contrast, glucagon-like peptide-1 (GLP-1) (satiety hormone) decreased after the UPF diet [33••]. These favourable changes in appetite-regulating gut hormones on the MPF diet compared with the UPF diet may explain the different energy intakes and weight change.

### Mechanisms: Discussion

So far, the effect of UPFs has not been explained by nutrients or food groups. Whilst UPFs tend to be nutrient-poor, some score well in nutrient profiling models such as Nutri-Score or UK multiple traffic lights [47, 86]. Indeed, adherence to the US Dietary Guidelines for Americans (US DGA) can be achieved with a > 90% proof-of-concept UPF diet, with a relatively low-energy density (0.9 kcal/g) and high healthy eating index score (86/100) [87]. But, whether this reflects a healthy UPF diet, or a major flaw in dietary guidance, is unclear [88]. There is a need for long-term, high-quality clinical trials assessing the effect of UPF vs. MPF, independent of factors already in dietary guidance. No such trial has been published, but an on-going randomised, controlled, free-living crossover trial is assessing the health impact of adhering to the UK dietary guidelines with MPF and UPF diets, with additional aims of understanding the effect of UPF on aspects of energy balance, including gut hormones, appetite and brain function (NCT05627570).

The relationships between food processing, texture, taste and energy density on energy intake are inconclusive. The few studies to date have been conducted in controlled laboratory settings, which, whilst necessary to manipulate and tightly measure food properties, may not incorporate important behavioural, social and environmental real-world influences on eating behaviour [89]. Only one study assessed intake for longer than 1 day, and it was not designed to examine mechanisms of UPF on energy intake. These trials tested a small selection of foods, usually as mixed meals. These were not always typical UPFs such as SSBs, breads, breakfast cereals or sweets. Texture, taste and energy density vary greatly within and across NOVA groups (e.g. the energy density of ASBs and SSBs or water and whole milk) [71]. Some authors therefore consider these properties to be independent of processing [45, 72] and argue that they should be controlled for [90]. Whilst texture and energy density are influenced by factors other than ultra-processing, they are also inherently linked with methods of ultra-processing. Extrusion moulding or use of gelling agents alters food texture and mouthfeel. UPFs tend to have a lower water content than MPFs and PFs, despite containing a similar nutrient content to PFs [47]. The lower water content is favourable for extending shelf-life, but also increases energy density. Considering properties that are fundamentally altered by ultra-processing as being independent, rather than overlapping or intrinsic to ultra-processing, diminishes the holistic concept of NOVA to whether a food contains additives or not. Trials (such as https://restructureproject.org, NCT05290064 and NCT05550818) are currently underway that will assess how texture, taste and energy density across a range of products and processing levels in the food environment influence energy intake. These trials will uncover the mechanisms of UPF driving excess energy intake and obesity [91].

## Methodology Appraisal: Is the Evidence Robust?

For confidence in the predominantly observational evidence to date, it is paramount that methods of estimating UPF are valid, accurate and consistent. There are suggestions that NOVA is not robust enough to classify foods into discrete processing categories [92, 93], with misclassification and coding disagreements between researchers [92].

One convenience sample of over 150 French food and nutrition specialists found poor agreement in coding foods into NOVA [94]. However, other studies show that good agreement is achieved with adequate training [24, 87, 95–97]. In several US cohorts, three independent researchers reached 95.6% coding agreement on food items [95]. In another, 88.3% agreement was reached for over 3000 foods [97]. One study even found greater agreement between coders for classifying foods into NOVA, than agreement on whether these foods could fit within a diet meeting the US DGA [87].

Assumptions made on the level of processing may lead to misclassification. Some studies address this with a sensitivity analysis, using more or less conservative approaches to assign foods to NOVA groups, and/or using different reference information from dietary reports or nutrient databases [24, 96]. In the US National Health and Nutrition Examination Survey, only 8% of items were assigned a different NOVA classification in a sensitivity analysis. Minimum and maximum estimates of UPF intake ranged from 53.4 to 60.1%, indicating similar estimates, regardless of the approach or assumptions [24]. In three US cohorts, only 5–10% of items were flagged for sensitivity analysis [95].

Coding differences between studies may also reflect cultural differences in food processing, not disagreement. Differing use of additives, preservatives or production methods across countries can classify a product as UPF in one country, but MPF/PF in another. In Australia, packaged breads are typically PF [98], whereas most UK breads are UPF [99]. To account for this, one multi-national cohort assigned country-specific NOVA groups for each food item [96].

A concern with epidemiological studies has been classifying foods into NOVA using food frequency questionnaires (FFQs) [90], rather than diet recalls or diaries, which offer greater detail. In studies adjusting for diet quality (Table 2), 9/17 relied solely on FFQs. However, observational studies rank participants to compare disease risk across quantiles of an exposure, rather than identifying exact exposures. Compared with recalls or diet records, FFQs show good agreement in ranking participants into quantiles of UPF intake [100–105]. Furthermore, several diet assessments including generic and NOVA-specific FFQs have been validated for classifying according to NOVA [100–104]. Correlations between NOVA-specific FFQs [102, 104] and diet records or multiple 24-h recalls are comparable to the correlations of nutrient intakes obtained from FFQs (*r* ~ 0.45–0.70), with reproducibility of 0.5–0.7 [106]. Moreover, a NOVA-specific, web-based 24-h recall showed good-moderate agreement with an interviewer-led 24-h recall for estimating NOVA groups, and substantial-near-perfect agreement in classifying participants into quintiles [107]. UPF intake has also been validated against or associated with biomarkers of processing or nutrient intake [108] (Supplementary Materials). However, biomarker studies are limited in validating the wider characteristics of UPFs given their heterogeneous characteristics.

Current food- and nutrient-based national dietary guidelines have been developed based on evidence predominantly from cohort studies rather than RCTs [109], with subsequent trials supporting the observation-based recommendations. Indeed, cohort studies demonstrate similar directions of association and consistency as RCTs with matched populations, intervention/exposures, comparator and outcomes in nutrition research [110, 111]. This highlights the value of well-designed cohort studies in assessing diet-disease relationships and generating public health dietary recommendations. However, despite a similar level of evidence for UPF, there is a call for further randomised controlled trials to address methodological limitations [112, 113].

### Methodology Appraisal: Discussion

NOVA is not perfect, but it has demonstrated utility as a tool to identify novel exposure-outcome associations, beyond current understanding of diet and obesity. Good interrater agreement is achieved with sufficient training, where most items are confidently and consistently classified. A priority should be agreement across stakeholders on how to define and measure ultra-processed food, with development of consensus guidelines. Until then, researchers should utilise papers reporting coding procedures for 24-h recalls [24] and FFQs [95], best-practice for applying NOVA [114], and decision flowcharts to simplify the coding process [24]. Machine learning may automate classification in the future [115]. Coding agreement between researchers should be reported, and for the small number of items with uncertainty in classification, sensitivity analyses should report minimum and maximum estimates (with confidence intervals) of UPF intake, repeating exposure-outcome analyses with these estimates. One multi-national cohort repeated their analysis with minimum and maximum estimates, which did not alter findings of increased body weight [36]. Further work to validate UPF intake from generic diet assessment tools (FFQs, food diaries, interviewer-led recalls) will strengthen confidence in existing reports. NOVA-specific tools should be developed and validated for each country, given cultural differences in food choice and food production methods.

## Time to Act on UPF?

UPF intake increases the risk of weight gain, overweight and obesity. From a public health standpoint, the precautionary principle is warranted. Individuals with the capacity to do so should be supported to reduce their intake, whilst acknowledging the role of UPFs for at-risk groups (e.g. food security and nutrient fortification, even though MPFs can be fortified, such as flour with iron) [41]. Current public dietary guidance provides an important framework for reducing disease and improving health. The holistic concept of food processing will not displace this. Rather, evidence regarding UPF should complement and expand current understanding of diet and obesity, with frameworks incorporating NOVA into national dietary guidelines having been published [116]. Several countries now incorporate UPF into their dietary guidelines [117, 118], as well as UNICEF, WHO [119, 120] and PAHO [121] in their guidance for overweight/obesity and health. However, the value of including UPF into dietary guidelines is still of scientific debate [122]. The role of UPF on obesity risk is a question for the 2025 US Dietary Guidelines Advisory Committee [123], and the UK Scientific Advisory Committee on Nutrition [113] and British Nutrition Foundation [112] concluded that there is insufficient evidence to include UPF within dietary guidelines. Thus, the research recommendations outlined in this review will strengthen the rationale for wider implementation of UPF into dietary guidelines.

### The Ultra-Processed Food System

The concept of NOVA has raised awareness of the upstream environmental drivers of obesity, shifting perspectives of diet and obesity away from individual choice and personal blame, and towards the food system dominated by trans-national, for-profit, UPF corporations (TNC) [124]. TNCs develop UPF products with the purpose of maximising consumption, to increase sales, and therefore, profit [125]. As a result, properties of UPFs that may promote overconsumption have been optimised over years of development, in a food industry that has been increasingly financially incentivised since the 1980s [125, 126]. Reductionist approaches that focus on nutrient reformulation alone or completely isolate food properties (e.g. texture, taste and energy density) from ultra-processing fail to acknowledge the environmental drivers of obesity and influence of TNCs [11].

Complete UPF avoidance should not be the goal, but the sociodemographics with the highest intakes should be supported to reduce consumption [23]. Furthermore, not all individuals have the capacity to reduce UPF intake. Thus, nutrient reformulation will remain a strategy for mitigating the harms of UPFs [41], whereby reformulation to lower energy density can support weight management by lowering energy intake [127, 128]. But, it must be considered as to what extent nutrient reformulation as the primary dietary obesity prevention strategy will solve the pandemic. A food is not the sum of its parts [129, 130], and reformulation does little to address synergistic aspects of food. Other aspects inherent to ultra-processing may have adverse impacts upon health, independent of nutrients. Reformulation also does not remove the original product from shelves; thus, the burden of choice remains with the individual. For TNCs, action to reformulate the adverse properties of UPFs is unlikely to be made voluntarily at the expense of profit, given the lack of action on nutrient reformulation to date and shareholder influence [125]. TNCs may develop nutrient reformulations with health claims for marketing, but how a TNC may produce other reformulations is unclear (e.g. texture reformulation), given the inherent alterations of food properties with ultra-processing.

The lens of NOVA and concept of UPF facilitates wider collective action regarding public health, sustainability, environment and agricultural policy, aligning stakeholders towards a common goal to change the food system. Local, national and international stakeholders must combine to address the obesogenic food system [131], which requires significant structural and regulatory changes [124, 126]. Multi-faceted policies, regulations, taxes and limits on UPF need to occur in tandem with development of accessible, subsidised and sustainable alternatives.

## Conclusions

Recent changes in the food environment, with greater access to and consumption of UPF has been a key driver of obesity. Observational evidence demonstrates positive associations between UPF, weight gain and obesity, and clinical evidence demonstrates increased energy intake and weight gain with UPFs. Mechanisms by which UPF may promote obesity are numerous, but inconclusive. UPFs may capture several characteristics that may encourage overconsumption, which may be insufficiently covered by most national dietary guidelines. The concept of UPF has strongly indicated the need for collective stakeholder action to change the obesogenic environment, giving individuals the agency to reduce their UPF consumption.

### Supplementary Information

Below is the link to the electronic supplementary material.

Supplementary file1 (DOCX 59 KB)

## Data Availability

No datasets were generated or analysed during the current study.
