# Supplementary material for: Ultra-processed Food and Obesity: What Is the Evidence?
Source: Curr Nutr Rep. 2024 Jan 31;13(1):23–38. doi: 10.1007/s13668-024-00517-z (PMC10924027; doi:10.1007/s13668-024-00517-z)
Supplement: Supplementary file 1 — Supplementary file1 (DOCX 59 KB) [file 13668_2024_517_MOESM1_ESM.docx]

**Supplementary Materials**

Numerous other prospective studies indicate increased risks of weight gain and obesity with increasing UPF consumption^1–12^, and in a dose-response manner^4,6,7,9,11,13–19^. Numerous other reviews and systematic reviews show similar findings, largely from the same evidence^20,20–27^. In children and adolescents, systematic reviews demonstrate increased risks of overweight, obesity, gestational weight gain and elevated WC with greater UPF intake ^28–31^. A small number of other child prospective studies do not suggest an increased risk^32–35^. UPF intake has been validated against or associated with biomarkers of processing or nutrient intake^36–41^. However, biomarker studies are limited in validating the wider content of UPFs, given the heterogeneous nature of UPFs.

**Supplementary Materials References:**

1 Magalhães EI da S, de Oliveira BR, Rudakoff LCS, de Carvalho VA, Viola PC de AF, Arruda SPM *et al.* Sex-Dependent Effects of the Intake of NOVA Classified Ultra-Processed Foods on Syndrome Metabolic Components in Brazilian Adults. *Nutrients* 2022; **14**: 3126.

2 Heerman WJ, Sneed NM, Sommer EC, Truesdale KP, Matheson D, Noerper TE *et al.* Ultra-processed food consumption and BMI-Z among children at risk for obesity from low-income households. *Pediatr Obes* 2023; **18**: e13037.

3 Handakas E, Chang K, Khandpur N, Vamos EP, Millett C, Sassi F *et al.* Metabolic profiles of ultra-processed food consumption and their role in obesity risk in British children. *Clin Nutr* 2022; **41**: 2537–2548.

4 Wang Y, Wang K, Du M, Khandpur N, Rossato SL, Lo C-H *et al.* Maternal consumption of ultra-processed foods and subsequent risk of offspring overweight or obesity: results from three prospective cohort studies. *BMJ* 2022; **379**: e071767.

5 Pang T, Gray HL, Alman AC, Buro AW, Basu A, Lu S *et al.* Ultra-processed food consumption and obesity indicators in individuals with and without type 1 diabetes mellitus: a longitudinal analysis of the prospective Coronary Artery Calcification in Type 1 Diabetes (CACTI) cohort study. *Public Health Nutr* 2023; **26**: 1626–1633.

6 Pan F, Wang Z, Wang H, Zhang J, Su C, Jia X *et al.* Association between Ultra-Processed Food Consumption and Metabolic Syndrome among Adults in China-Results from the China Health and Nutrition Survey. *Nutrients* 2023; **15**: 752.

7 González-Palacios S, Oncina-Cánovas A, García-de-la-Hera M, Martínez-González MÁ, Salas-Salvadó J, Corella D *et al.* Increased ultra-processed food consumption is associated with worsening of cardiometabolic risk factors in adults with metabolic syndrome: Longitudinal analysis from a randomized trial. *Atherosclerosis* 2023; **377**: 12–23.

8 Canhada SL, Vigo Á, Luft VC, Levy RB, Alvim Matos SM, del Carmen Molina M *et al.* Ultra-Processed Food Consumption and Increased Risk of Metabolic Syndrome in Adults: The ELSA-Brasil. *Diabetes Care* 2022; **46**: 369–376.

9 Tan L-J, Hwang SB, Shin S. The Longitudinal Effect of Ultra-Processed Food on the Development of Dyslipidemia/Obesity as Assessed by the NOVA System and Food Compass Score. *Mol Nutr Food Res* 2023; : e2300003.

10 Rudakoff LCS, Magalhães EI da S, Viola PC de AF, de Oliveira BR, da Silva Coelho CCN, Bragança MLBM *et al.* Ultra-processed food consumption is associated with increase in fat mass and decrease in lean mass in Brazilian women: A cohort study. *Front Nutr* 2022; **9**.https://www.frontiersin.org/articles/10.3389/fnut.2022.1006018 (accessed 20 Nov2023).

11 Santos FS dos, Steele EM, Costa C dos S, Gabe KT, Leite MA, Claro RM *et al.* Nova diet quality scores and risk of weight gain in the NutriNet-Brasil cohort study. *Public Health Nutr* 2023; **26**: 2366–2373.

12 Rauber F, Chang K, Vamos EP, da Costa Louzada ML, Monteiro CA, Millett C *et al.* Ultra-processed food consumption and risk of obesity: a prospective cohort study of UK Biobank. *Eur J Nutr* 2021; **60**: 2169–2180.

13 Konieczna J, Morey M, Abete I, Bes-Rastrollo M, Ruiz-Canela M, Vioque J *et al.* Contribution of ultra-processed foods in visceral fat deposition and other adiposity indicators: Prospective analysis nested in the PREDIMED-Plus trial. *Clin Nutr* 2021; **40**: 4290–4300.

14 Canhada SL, Luft VC, Giatti L, Duncan BB, Chor D, da Fonseca M de JM *et al.* Ultra-processed foods, incident overweight and obesity, and longitudinal changes in weight and waist circumference: the Brazilian Longitudinal Study of Adult Health (ELSA-Brasil). *Public Health Nutr* 2020; **23**: 1076–1086.

15 Cordova R, Kliemann N, Huybrechts I, Rauber F, Vamos EP, Levy RB *et al.* Consumption of ultra-processed foods associated with weight gain and obesity in adults: A multi-national cohort study. *Clin Nutr* 2021; **40**: 5079–5088.

16 Chang K, Khandpur N, Neri D, Touvier M, Huybrechts I, Millett C *et al.* Association Between Childhood Consumption of Ultraprocessed Food and Adiposity Trajectories in the Avon Longitudinal Study of Parents and Children Birth Cohort. *JAMA Pediatr* 2021; **175**: e211573.

17 Beslay M, Srour B, Méjean C, Allès B, Fiolet T, Debras C *et al.* Ultra-processed food intake in association with BMI change and risk of overweight and obesity: A prospective analysis of the French NutriNet-Santé cohort. *PLoS Med* 2020; **17**: e1003256.

18 Mendonça R de D, Pimenta AM, Gea A, de la Fuente-Arrillaga C, Martinez-Gonzalez MA, Lopes ACS *et al.* Ultraprocessed food consumption and risk of overweight and obesity: the University of Navarra Follow-Up (SUN) cohort study. *Am J Clin Nutr* 2016; **104**: 1433–1440.

19 Sandoval-Insausti H, Jiménez-Onsurbe M, Donat-Vargas C, Rey-García J, Banegas JR, Rodríguez-Artalejo F *et al.* Ultra-Processed Food Consumption Is Associated with Abdominal Obesity: A Prospective Cohort Study in Older Adults. *Nutrients* 2020; **12**: 2368.

20 Wang Z, Lu C, Cui L, Fenfen E, Shang W, Wang Z *et al.* Consumption of ultra-processed foods and multiple health outcomes: An umbrella study of meta-analyses. *Food Chem* 2024; **434**: 137460.

21 Zhang Y, Giovannucci EL. Ultra-processed foods and health: a comprehensive review. *Crit Rev Food Sci Nutr* 2022; **0**: 1–13.

22 Jardim MZ, Costa BV de L, Pessoa MC, Duarte CK. Ultra-processed foods increase noncommunicable chronic disease risk. *Nutr Res* 2021; **95**: 19–34.

23 Harb AA, Shechter A, Koch PA, St-Onge M-P. Ultra-processed foods and the development of obesity in adults. *Eur J Clin Nutr* 2023; **77**: 619–627.

24 Louzada ML da C, Costa CDS, Souza TN, Cruz GL da, Levy RB, Monteiro CA. Impact of the consumption of ultra-processed foods on children, adolescents and adults’ health: scope review. *Cad Saude Publica* 2022; **37**: e00323020.

25 de Araújo TP, de Moraes MM, Magalhães V, Afonso C, Santos C, Rodrigues SSP. Ultra-Processed Food Availability and Noncommunicable Diseases: A Systematic Review. *Int J Environ Res Public Health* 2021; **18**: 7382.

26 Chen X, Zhang Z, Yang H, Qiu P, Wang H, Wang F *et al.* Consumption of ultra-processed foods and health outcomes: a systematic review of epidemiological studies. *Nutr J* 2020; **19**: 86:1-86;10.

27 Silva Meneguelli T, Viana Hinkelmann J, Hermsdorff HHM, Zulet MÁ, Martínez JA, Bressan J. Food consumption by degree of processing and cardiometabolic risk: a systematic review. *Int J Food Sci Nutr* 2020; **71**: 678–692.

28 Petridi E, Karatzi K, Magriplis E, Charidemou E, Philippou E, Zampelas A. The impact of ultra-processed foods on obesity and cardiometabolic comorbidities in children and adolescents: a systematic review. *Nutr Rev* 2023; : nuad095.

29 De Amicis R, Mambrini SP, Pellizzari M, Foppiani A, Bertoli S, Battezzati A *et al.* Ultra-processed foods and obesity and adiposity parameters among children and adolescents: a systematic review. *Eur J Nutr* 2022; **61**: 2297–2311.

30 Frías JRG, Cadena LH, Villarreal AB, Piña BGB, Mejía MC, Cerros LAD *et al.* Effect of ultra-processed food intake on metabolic syndrome components and body fat in children and adolescents: A systematic review based on cohort studies. *Nutrition* 2023; **111**: 112038.

31 Oliveira PG de, Sousa JM de, Assunção DGF, Araujo EKS de, Bezerra DS, Dametto JF dos S *et al.* Impacts of Consumption of Ultra-Processed Foods on the Maternal-Child Health: A Systematic Review. *Front Nutr* 2022; **9**.https://www.frontiersin.org/articles/10.3389/fnut.2022.821657 (accessed 22 Nov2023).

32 Vilela S, Magalhães V, Severo M, Oliveira A, Torres D, Lopes C. Effect of the food processing degree on cardiometabolic health outcomes: A prospective approach in childhood. *Clin Nutr* 2022; **41**: 2235–2243.

33 Whyte K, Contento I, Wolf R, Guerra L, Martinez E, Pi-Sunyer X *et al.* A Secondary Analysis of Maternal Ultra-processed Food Intake in Women with Overweight Or Obesity and Associations with Gestational Weight Gain and Neonatal Body Composition Outcomes. *J Mother Child* 2021; **25**: 244.

34 Pereyra González I, Farías-Antúnez S, Buffarini R, Gómez Ayora A, Fletcher AM, Rodrigues Domingues M *et al.* Ultra-processed food consumption and the incidence of obesity in two cohorts of Latin-American young children: A longitudinal study. *J Pediatr Nurs* 2023; **69**: e120–e126.

35 Lourenço BH, Castro MC, Sato P de M, Neves PAR, Vivanco E, Lima DL *et al.* Exposure to ultra-processed foods during pregnancy and ultrasound fetal growth parameters. *Br J Nutr* 2023; **130**: 2136–2145.

36 Martínez Steele E, Khandpur N, Louzada ML da C, Monteiro CA. Association between dietary contribution of ultra-processed foods and urinary concentrations of phthalates and bisphenol in a nationally representative sample of the US population aged 6 years and older. *PLOS ONE* 2020; **15**: e0236738.

37 Buckley JP, Kim H, Wong E, Rebholz CM. Ultra-processed food consumption and exposure to phthalates and bisphenols in the US National Health and Nutrition Examination Survey, 2013–2014. *Environ Int* 2019; **131**: 105057.

38 Huybrechts I, Rauber F, Nicolas G, Casagrande C, Kliemann N, Wedekind R *et al.* Characterization of the degree of food processing in the European Prospective Investigation into Cancer and Nutrition: application of the Nova classification and validation using selected biomarkers of food processing. *Front Nutr* 2022; **9**.https://www.frontiersin.org/articles/10.3389/fnut.2022.1035580 (accessed 29 Jun2023).

39 Stratakis N, Siskos AP, Papadopoulou E, Nguyen AN, Zhao Y, Margetaki K *et al.* Urinary metabolic biomarkers of diet quality in European children are associated with metabolic health. *eLife* 2022; **11**: e71332.

40 Amorim NCM, Silva AGCL da, Rebouças AS, Bezerra DS, Lima MSR, Medeiros JFP *et al.* Dietary share of ultra-processed foods and its association with vitamin E biomarkers in Brazilian lactating women. *Br J Nutr* 2022; **127**: 1224–1231.

41 Kelsey PT, Papadopoulou E, Borge TC, Dahl C, Brantsæter AL, Erlund I *et al.* Ultra-processed food consumption and associations with biomarkers of nutrition and inflammation in pregnancy: The Norwegian Environmental Biobank. *Front Nutr* 2022; **9**.https://www.frontiersin.org/articles/10.3389/fnut.2022.1052001 (accessed 22 Nov2023).
